# A novel field-based molecular assay to detect validated artemisinin-resistant *k13* mutants

**DOI:** 10.1186/s12936-018-2329-y

**Published:** 2018-04-24

**Authors:** Laurence Vachot-Ganée, Nimol Khim, Alexandra Iannello, Eric Legrand, Saorin Kim, Rotha Eam, Chanra Khean, Malen Ken, Elizabeth Ashley, Kyaw Myo Tun, Mehul Dhorda, François Nosten, Issa Mahamat Souleymane, Sophie Blein, Alexandre Pachot, Frédéric Ariey, Karine Kaiser, Didier Ménard

**Affiliations:** 1 Medical Diagnostic Discovery Department (MD3), bioMérieux, Grenoble and Marcy l’Etoile, France; 2 Malaria Molecular Epidemiology Unit, Institute Pasteur in Cambodia, Phnom Penh, Cambodia; 3 Genetics and Genomics of Insect Vectors Unit, Institute Pasteur, Paris, France; 4 Malaria Genetics and Resistance Group, Biology of Host-Parasite Interactions Unit, Institute Pasteur, Paris, France; 5 Mahidol–Oxford Tropical Medicine Research Unit, Bangkok, Thailand; 6 Department of Preventive & Social Medicine, Defence Services Medical Academy, Yangon, Myanmar; 7 Myanmar Oxford Clinical Research Unit, Yangon, Myanmar; 8 Worldwide Antimalarial Resistance Network, Asia Regional Centre, Bangkok, Thailand; 9 Centre for Tropical Medicine and Global Health, Nuffield Department of Medicine Research Building, University of Oxford, Old Road Campus, Oxford, UK; 10 Shoklo Malaria Research Unit, Mahidol-Oxford Tropical Medicine Research Unit, Faculty of Tropical Medicine, Mahidol University, Mae Sot, Thailand; 11 Programme National de Lutte Contre le Paludisme au Tchad, Ndjamena, Chad; 12 Institute Cochin Inserm U1016, Université Paris-Descartes, Sorbonne Paris Cité, Paris, France; 13 Laboratoire de Parasitologie-Mycologie, Hôpital Cochin, Paris, France

**Keywords:** Malaria, *Plasmodium falciparum*, Artemisinin resistance, *k13* mutation detection, Surveillance

## Abstract

**Background:**

Given the risk of artemisinin resistance spreading from the Greater Mekong sub-region, prospective monitoring in sub-Saharan Africa should be expedited. Molecular biology techniques used for monitoring rely on the detection of *k13* validated mutants by using PCR and Sanger sequencing approach, usually not available in malaria endemic areas.

**Methods:**

A semi-automated workflow based on the easyMAG^®^ platform and the Argene Solution^®^ (bioMérieux, Marcy l’Etoile, France) as a field-based surveillance tool operable at national level was developed in four steps. Clinical and analytical performances of this tool detecting five of the most frequent and validated *k13* mutants (Y493H, I543T, R539T, F446I and C580Y) from dried blood spots (DBS) were compared to the gold standard approach (PCR and Sanger sequencing).

**Results:**

By using the ARMS (amplification-refractory mutation system) strategy, the best multiplexing options were found in 3 separate real-time PCR duplexes (IC as internal control/I543T, C580Y/Y493H and F446I/R539T) with limits of detection ranging from 50 (C580Y) to 6.25 parasites/µL (Y493H). In field conditions, using 642 clinical DBS (from symptomatic patients and asymptomatic individuals) collected from Cambodia, Myanmar and Africa (Chad), the overall sensitivity and specificity of the K13 bMx prototype assay developed by bioMérieux were ≥ 90%. Areas under the ROC curves were estimated to be > 0.90 for all *k13* mutants in samples from symptomatic patients.

**Conclusion:**

The K13 ready-to-use bMx prototype assay, considered by the end-users as a user-friendly assay to perform (in shorter time than the K13 reference assay) and easy to interpret, was found to require less budget planning and had fewer logistical constraints. Its excellent performance qualifies the prototype as a reliable screening tool usable in malaria endemic countries recognized to be at risk of emergence or spread of validated *k13* mutants. Additional multi-site studies are needed to evaluate the performances of the K13 bMx prototype assay in different epidemiological contexts such as Africa, India, or South America.

**Electronic supplementary material:**

The online version of this article (10.1186/s12936-018-2329-y) contains supplementary material, which is available to authorized users.

## Background

In 2016, an estimated 216 million (196–263 million) cases of malaria and 445,000 deaths were recorded worldwide, mainly in sub-Saharan Africa (90%), followed by Southeast Asia (3%) and Eastern Mediterranean region (2%) [1]. Between 2010 and 2016, the incidence rate of malaria decreased by 18% globally [1]. This trend is commonly attributed to the widespread implementation of artemisinin-based combination therapy (an estimated 409 million treatment courses of ACT were procured in 2016) and, therefore, to the improvement of the management of falciparum uncomplicated malaria cases [2]. ACT combined a potent and fast-acting artemisinin derivative that reduces rapidly the parasite biomass with a long half-life partner drug that eliminates any remaining parasites [3].

Unfortunately, in 2007–2008, partial resistance to artemisinin derivatives (ART-R) was detected in Southeast Asia (first in Cambodia and later in Myanmar, Thailand, Vietnam and Lao PDR) followed more recently by the emergence of resistance to piperaquine (PPQ-R) [4–6]. At present, the reduced efficacy of ART and PPQ translates into late treatment failures and prolonged parasite carriage, thereby increasing the transmission potential of multidrug resistant infections [7–11]. Dihydroartemisinin–piperaquine (DHA–PPQ) treatment failures are estimated to reach 60% in some Cambodian provinces, indicating the dramatic spread of both ART-R and PPQ-R across Cambodia and beyond. Indeed, recent genomic investigations have demonstrated that *Plasmodium falciparum* ART-R and PPQ-R have spread from western Cambodia to neighbouring provinces in Cambodia and then to north-eastern Thailand, southern Laos and southern Vietnam, leading to a substantial increase in DHA–PPQ failure rates. This rapid expansion was clearly shown to be associated to the hard-selective sweep of a single fit ART-R *P. falciparum* parasite lineage, outcompeting the other resistant malaria parasites, and subsequently acquiring PPQ-R [9, 12].

Since 2014, non-synonymous mutations in the propeller domain of a *kelch* gene located on the chromosome 13 (*k13*, PF3D7_1343700) have been shown to be a major determinant of ART-R [13–15]. Currently, the molecular surveillance of the global extent of ART-R malaria parasites relies on these markers [16]. To date, almost 200 *k13* mutations have been described worldwide. In Southeast Asia, *k13* mutants are frequent and distributed between Cambodia–Vietnam–Lao PDR (C580Y, R539T, Y493H and I543T) and western Thailand–Myanmar–China (C580Y, F446L, N458Y, P574L and R561H) with almost no overlap (except C580Y and P553L which has been found in the two areas) [17]. The C580Y mutant is largely predominant in Southeast Asia. In Africa, a broad array of rare non-synonymous mutations is observed at very low frequency. Clinical and biological investigations have shown that not all non-synonymous *k13* mutations confer ART-R [17]. To be validated as ART-R molecular marker, a *k13* mutant has to be correlated with delayed parasite clearance in clinical studies and reduced drug in vitro susceptibility (survival rate ≥ 1% expressed by the ring-stage survival assay, RSA^0–3h^) in fresh isolates (ex vivo assays) or culture-adapted field parasites or K13 genome-edited parasites (in vitro assays) [18]. According to the latest WHO update on artemisinin resistance (released in April 2017), only six *k13* mutants are validated (C580Y, Y493H, R539T, I543T, N458Y, R561H) [16]. The F446I mutant, which is highly prevalent in Myanmar, is strongly suspected of being associated to ART-R [17, 19–22].

In such an epidemiological context, the risk of ART-R parasites spreading from the Greater Mekong sub-region (GMS) to Africa, as happened previously with chloroquine and sulfadoxine/pyrimethamine-resistant parasites, is a major concern threatening the world’s malaria elimination efforts [23]. This alarming situation mandates close surveillance and containment of the dissemination of ART-R parasites in Southeast Asia, but above all implementation of proactive monitoring of anti-malarial drug resistance in sub-Saharan Africa. The emergence and the spread of ART-R outside Southeast Asia can now be easily tracked in real-time by the detection of *k13* validated mutants in dried blood spots (DBS) collected from finger prick [24]. Molecular biology techniques used for ART-R monitoring rely mainly on polymerase chain reaction (PCR) and Sanger sequencing. However, sequencing platforms are not available in most malaria endemic areas, thus obligating the shipment of the samples or the PCR products to appropriate facilities. Substantial work remains to be done to harmonize and validate collected data. This includes the development of field-based molecular biology techniques, the provision of control samples, or the implementation of a quality assurance system [25].

To overcome this issue, a semi-automated workflow based on the easyMAG^®^ platform and the Argene Solution^®^ (bioMérieux, Marcy l’Etoile, France) as a field-based surveillance tool operable at national level was developed and evaluated. This tool (referred as K13 bMx prototype) allows the detection of the five most frequent and validated ART-R *k13* mutants from DBS and can be deployed in most laboratories in malaria endemic areas easily (Additional file 1). The clinical and analytical performances of the K13 bMx prototype were compared to the gold standard approach (QiaAmp DNA blood mini kit extraction followed by PCR Sanger sequencing referred to as K13 reference assay) [15] and are presented in this manuscript.

## Methods

### K13 bMx prototype: development and validation

The prototype was developed in four sequential steps.

*Step 1* The easyMAG^®^ DNA extraction protocol from DBS was optimized. First, a new format of 3 MM filter paper was designed to facilitate the semi-automated extraction protocol and avoid inter-sample DNA contamination (Additional file 2). Second, the limit of detection (LoD), the repeatability and the reproducibility of the easyMAG^®^ DNA extraction protocol were assessed by using artificial DBS (prepared from the 3D7 culture-adapted line at parasite densities ranging from 5 to 0.6 parasite/µL). Real-time PCR assay targeting the *cytochrome c oxidase subunit 1* gene (PlasmoDB gene ID: mal_mito_2 cytochrome c oxidase subunit 1) was used to evaluate the extraction efficiency. This PCR assay was thereafter integrated as internal control (IC) in the K13 bMx prototype. Third, results of real-time PCR assay targeting the *Plasmodium cytochrome b* gene (PlasmoDB gene ID: mal_mito_3 cytochrome b) using DNA extracted with the QiaAmp DNA blood mini kit (Qiagen, Hilden, Germany) and real-time PCR assay targeting the *cytochrome c oxidase subunit 1* gene using DNA extracted with the easyMAG^®^ DNA extraction protocol were compared. DNA were obtained from *P. falciparum* blood samples (clinical *falciparum* malaria cases with parasitaemia ranging from 0.1 to 2% and asymptomatic individuals harbouring submicroscopic *P. falciparum* PCR positive infection with a parasite density < 0.01%) as well as negative blood samples spotted onto filter paper (50 µL of blood).

*Step 2* The analytical performances of single and multiplex real-time PCR assays designed to detect C580Y, Y493H, R539T, I543T, F446I *k13* mutants (PlasmoDB gene ID: PF3D7_1343700) and the *cytochrome c oxidase subunit 1* gene (used as internal control, IC) were assessed. First, in silico strategies were used to design specific PCR primers. Second, single and then multiplex real-time PCRs for each primer set were optimized to reach the best analytical performances (LoD) by using gDNA extracted from *P. falciparum* culture-adapted isolates harbouring different *k13* alleles (wild-type, C580Y, Y493H, R539T, I543T, F446I, parasite densities ranging from 4000 to 0  parasites/µL) or from DBS containing 50 µL blood of *P. falciparum* culture-adapted isolates containing decreasing parasite densities of each *k13* mutant (from 100 to 6.25 parasites/µL) extracted with the easyMAG^®^.

*Step 3* The performances of the K13 bMx prototype (from DBS to results) were assessed at bioMerieux (Grenoble, France). The LoD, repeatability and reproducibility were then evaluated by using artificial DBS (obtained from wild-type and *k13* mutants culture-adapted lines at parasite densities ranging from 500 to 6.25 parasites/µL). The specificity (absence of cross reactivity with other *Plasmodium* species) was assessed by using pure DNA fro*m P. vivax, Plasmodium ovale* and *Plasmodium malariae* (from DBS at 4000, 100 and 10 parasites/µL per species) or DNA mixed at the same parasite densities with *P. falciparum* 3D7 DNA (4000 parasites/µL). Finally, the performances were evaluated with DBS collected in Cambodia and Myanmar from malaria cases (symptomatic patients and asymptomatic individuals) or malaria-free individuals. K13 reference assay used as gold standard method was performed at Institute Pasteur Cambodia (IP Cambodia, Phnom Penh, Cambodia) on the same specimens. The technicians were blinded to results from the other method.

*Step 4* The final performances of the K13 bMx prototype used in field conditions were evaluated at IP Cambodia. Tested DBS samples were obtained in Cambodia, Myanmar and Africa from symptomatic patients, asymptomatic and malaria-free individuals. Molecular tests (K13 bMx prototype and K13 reference assays) were performed independently by technicians with no knowledge of each other’s results.

### Samples

For steps 1, 2 and 3, a set of *P. falciparum* isolates harbouring different *k13* alleles (C580Y, Y493H, R539T, I543T, F446I) and the 3D7 reference line (*k13* wild-type) were adapted for long-term in vitro culture. Culture adaptation of the field Cambodian isolates was performed as described previously [26]. Artificial DBS specimens, mimicking a whole-blood sample obtained from patients, were prepared from culture-adapted parasites by spotting 50 µL blood (50% haematocrit) with decreasing parasite densities of each *k13* wild-type and mutants onto the 3 MM filter paper. Parasite-free blood samples were obtained from the blood bank (Phnom Penh, Cambodia) and were used to prepare artificial malaria-free DBS.

For steps 1, 3 and 4, blood samples (284 and 642 clinical DBS for steps 3 and 4, respectively) were obtained from febrile patients suspected of having malaria and seeking anti-malarial treatment in health facilities or in villages (through the village malaria workers network) across Cambodia, Myanmar and Chad. Malaria diagnosis was achieved in the field either by microscopy of Giemsa-stained malaria blood films or by malaria rapid diagnostic test (CareStart Malaria HRP2/pLDH Pf/PAN Combo, Access Bio, USA) detecting both *P. falciparum* and non-*P. falciparum* infections. After obtained written informed consent, DBS were prepared from finger prick (using the new format of 3 MM filter paper or the Whatman FTA card). Fresh capillary blood samples spotted onto the 3 MM filter paper were obtained from asymptomatic individuals living in villages in Rattanakiri and Preah Vihear province (Cambodia) during cross sectional surveys conducted in 2016–2017 as previously described [27, 28].

### Parasite DNA extraction

Parasite DNA was extracted at IP Cambodia from red blood cell pellets obtained from culture-adapted isolates using the QiaAmp DNA blood mini kit (Qiagen) following manufacturer’s recommendations. Parasite DNA was extracted from clinical or artificial DBS using the QiaAmp DNA blood mini kit (Qiagen) at IP Cambodia or the easyMAG^®^ system in both sites (IP Cambodia and bioMérieux), according to the supplier’s recommendations. DNA obtained from the easyMAG^®^ Generic 2.0.1 protocol was extracted using 2 mL lysis buffer with 50 µL of silica and eluted in a volume of 50 µL.

### Detection of Plasmodium parasites and *k13* non-synonymous mutants

At IP Cambodia, *Plasmodium* DNA was detected using a two-step real-time PCR assay targeting the *Plasmodium cytochrome b* gene, as previously described [15]. Briefly, each blood sample was screened using genus-specific primers for malaria infection. Only positive samples were tested for *Plasmodium* species identification using species-specific primers for the four main human malaria species (*Plasmodium vivax, P. falciparum, P. ovale*, *P. malariae*). In the K13 reference assay, amplification by nested-PCR of the K13-propeller domain (codons 443–666, i.e. 720 bp) was used to detect *k13* mutations by sequencing, according to the operating procedures previously developed [15, 17]. PCR products were sequenced by Macrogen (Seoul, South Korea). Electrophoregrams were analysed on both strands with CEQ 2000 genetic analysis system software (Beckman Coulter, Villepinte, France), using PF3D7_1343700 as reference sequence. Isolates with mixed alleles were considered as mutants.

In the K13 bMx prototype assay, *P. falciparum* DNA (IC, targeting the *cytochrome c oxidase subunit 1* gene) and *k13* mutants (C580Y, Y493H, R539T, I543T, F446I) were detected in 3 separate real-time PCR duplexes (IC/I543T, C580Y/Y493H and F446I/R539T) using FAM and HEX fluorophores. Real-time PCR duplexes reactions were done with 15 μL of ready-to-use reaction mix and 10 μL of DNA extract and amplified on the CFX96 PCR instrument (Bio-Rad, Marnes-la-Coquette, France). PCR amplifications were performed under the following conditions: heating at 95 °C for 15 min, followed by 45 cycles of heating at 95 °C for 10 s, and of annealing/extension at 60 °C for 40 s. For each run, positive (gDNA from culture-adapted Cambodian *P. falciparum* parasites harbouring each of the *k13* mutant alleles and from the 3D7 line) and no template (elution buffer) controls were included.

The “automatic threshold mode” was applied to define Cq and fluorescence values (usually set up at ~ 10% of the average fluorescence). A specimen was considered positive for the tested mutation if the delta Cq (Cq mutant PCR–Cq IC PCR) was below a specific cut-off. This cut-off was set at 6 for I543T mutant, 9 for R539T and F446 mutants and 11 for Y493H and C580Y mutants.

### Statistical analysis

Data were recorded and analysed using Excel software and MedCalc (MedCalc Software, Belgium). The F-test was used to compare variances of Cq values.

To assess the analytical performance of the K13 bMx prototype assay (vs. the K13 reference assay), standard diagnostic test measures were determined. Sensitivity (Se) was the proportion of DBS that the K13 bMx prototype assay classified as mutant among DBS classified as *k13* mutant (C580Y, Y493H, R539T, I543T, or F446I) with the K13 reference assay (true positive rate). Specificity (Sp) was the proportion of DBS that the K13 bMx prototype assay classified as *k13* non-mutant (C580Y, Y493H, R539T, I543T, or F446I) among DBS classified as *k13* non-mutant (included in the panel assay) with the K13 reference assay (true negative rate). Positive predictive value (PPV) was the probability that a *k13* mutant included in the panel assay was present in DBS when the K13 bMx prototype assay classified DBS as *k13* mutant and negative predictive value (NPV), the probability that a *k13* mutant included in the panel assay was not present in DBS when the K13 bMx prototype assay classified DBS as no mutant.

PPV and NPV were calculated based on the proportion of *k13* mutants (C580Y, Y493H, R539T, I543T, or F446I, referred as PP) found in the set of tested DBS as following:

$$ {\text{PPV}}\, = \,{\text{Se}} \times {\text{PP}}/{\text{Se }}\times {\text{PP}}\, + \,\left( { 1- {\text{Sp}}} \right) \times \left( { 1- {\text{PP}}} \right){\text{ and NPV}}\, = \,{\text{Sp}} \times \left( { 1- {\text{PP}}} \right)/{\text{Sp}} \times \left( { 1- {\text{PP}}} \right)\, + \,\left( { 1- {\text{Se}}} \right) \times {\text{PP}}. $$

The AURoC values (Area under a ROC curve) were estimated to assess the accuracy of the K13 bMx prototype assay to detect the five *k13* mutants included in the panel, based on the results of the K13 reference assay. An AUC value of 1 represents a perfect test (i.e. a test that would classify all screened mutants exactly as the reference test) and an AUC value of 0.5 represents a worthless test. AUC value > 0.90 was considered to represent a diagnostic test with an excellent accuracy.

The 95% confidence intervals (CI 95%) for sensitivity and specificity are “exact” Clopper–Pearson confidence intervals. CI 95% for the PPV and NPV are the standard logit confidence intervals given by Mercaldo et al. [29]. The CI 95% for AUC values were used to test the hypothesis that the theoretical area is 0.5. All reported *P*-values are two-sided and were considered statistically significant if < 0.05.

## Results

### Performances of the easyMAG^®^ extraction protocol (step 1)

**Fig. 1:**
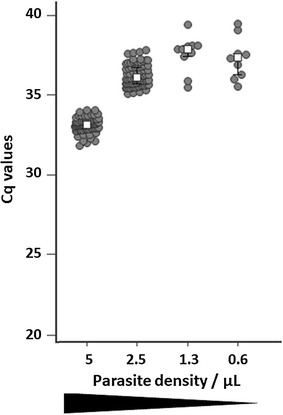
Performances of the easyMAG^®^ extraction protocol: Cq values from real-time PCR assay targeting the *cytochrome c oxidase subunit 1* gene with DNA extracted from artificial DBS (3D7 line at 5–0.6 parasites/µL)

The Cq values obtained from artificial DBS (3D7 line at 5–0.6 parasites/µL) are presented in Fig. 1. The LoD of the real-time PCR assay targeting the *cytochrome c oxidase subunit 1* gene using DNA extracted with the easyMAG^®^ DNA extraction protocol was < 0.6 parasite/µL. Runs assessing the reproducibility and the repeatability showed no significant difference of the median Cq values according to the operators (N = 2, P = 0.47, F-test), batches of reagents (N = 2, P = 0.93, F-test) or the easyMAG^®^ system (N = 2, P = 0.73, F-test).

Seventy clinical DBS were tested both at IP Cambodia (using QiaAmp DNA blood mini kit Qiagen/two-step real-time PCR assays targeting the *Plasmodium cytochrome b* gene) as previously described [30, 31] and at bioMérieux (using the easyMAG^®^ system and the real-time PCR targeting the *cytochrome c oxidase subunit 1* gene). The proportion of concordant results between both methods was very high (64/70 DBS, 91%), more frequent with DBS collected from symptomatic patients (29/30 DBS, 97%) than DBS collected from asymptomatic individuals (35/40 DBS, 88%) (Additional file 3). Six samples were found to have discordant results: one asymptomatic case was detected as *P. falciparum* infection at IP Cambodia and negative at bioMérieux; three asymptomatic cases were detected as negative at IP Cambodia and as *P. falciparum* infection at bioMérieux and two cases (one symptomatic and one asymptomatic) were detected as *P. vivax* infections at IP Cambodia and *P. falciparum* infections at bioMérieux.

### Development of multiplex real-time PCR assays to detect ART-R validated *k13* mutations (step 2)

Two molecular strategies were explored to detect the presence of SNPs (single nucleotide polymorphisms): TaqMan using mutant specific probes and Amplification-refractory mutation system (ARMS). The ARMS technique, based on allele-specific primers [32] (Additional file 4) was found to be the best option (high multiplexing capacity). First, allele-specific ARMS primers were designed and tested to detect each *k13* mutations in simplex reactions. Second, multiplex strategies by mixing simplex reactions were tested and optimized (testing different concentrations of MgCl_2_, dNTP and primers/probe) using gDNA extracted from *k13* wild-type and mutant culture-adapted *P. falciparum* isolates. The best analytical performances were observed in 3 separate real-time PCR duplexes (IC/I543T, C580Y/Y493H and F446I/R539T) using FAM and HEX fluorophores, as presented in Table 1.

**Table 1:**
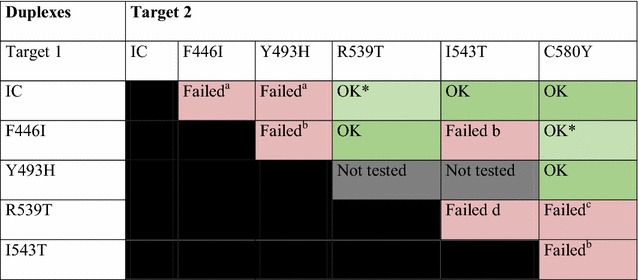
Summary of the results obtained for the multiplex strategies by mixing simplex reactions

* Of note, similar performances with those selected were obtained with 446/580 and IC/539 duplexes, demonstrating the multiplexing flexibility of the ARMS strategy

Failed means: ^a^ decrease of sensitivity to detect both SNPs, ^b^ presence of non-specific amplification curves, ^c^ decrease of sensitivity to detect one SNP (R539T), ^d^ overlap of the primers set

### Analytical performances of the K13 bMx prototype assay (from DBS to results) performed in controlled condition (step 3)

**Fig. 2:**
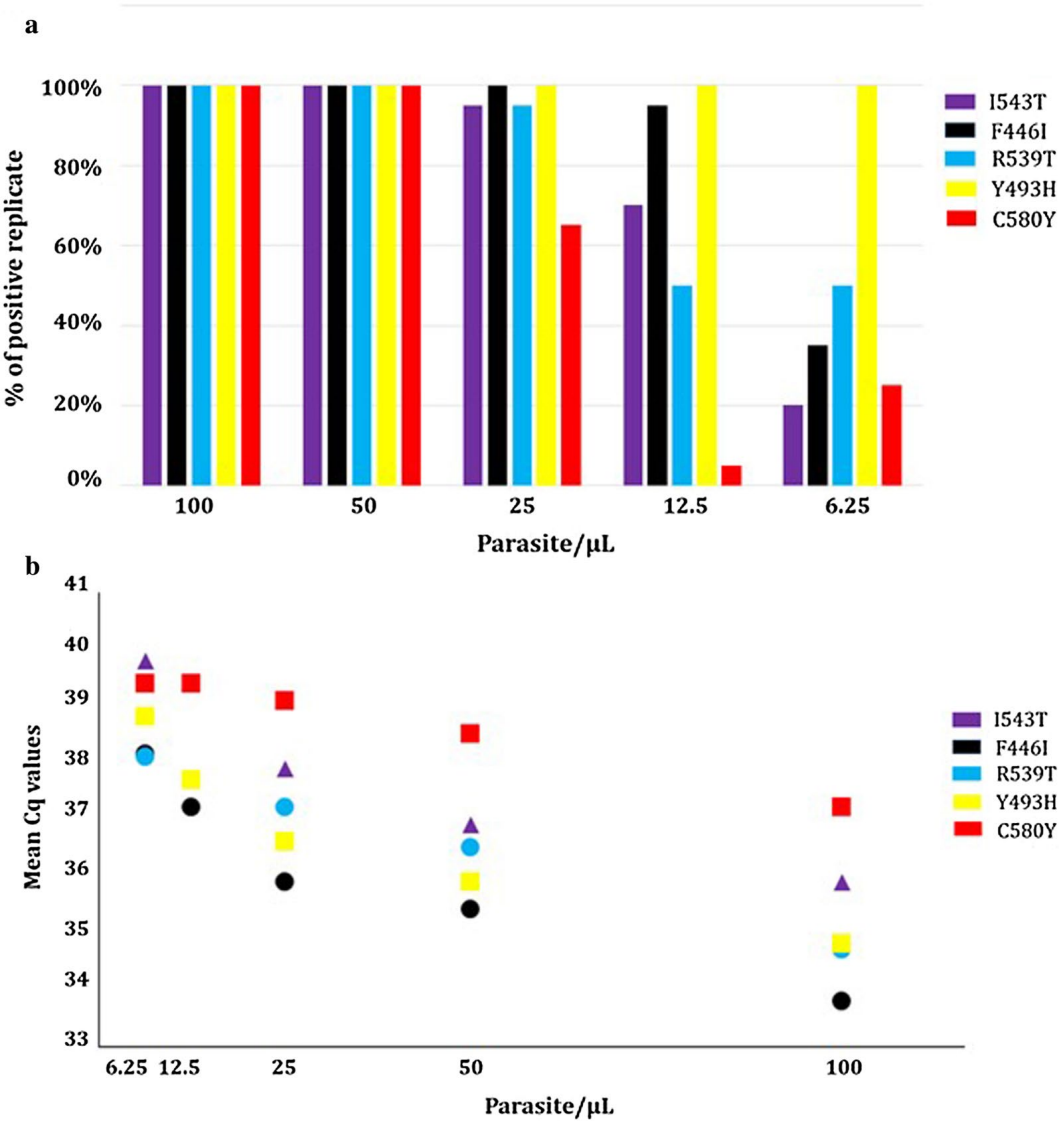
**a** Limits of detection (% of positive replicates, 20 replicates per parasite density for K13 mutant) of each duplex obtained from DNA extracted by the easyMAG^®^ system (Generic 2.0.1 protocol) from artificial DBS containing decreasing parasite densities of each K13 mutant (from 100 to 6.25 parasites/µL). **b** Mean Cq values determined according to the parasite density of *k13* mutant (from 100 to 6.25 parasites/µL)

No signal of amplification was obtained with pure *P. vivax, P. ovale, P. malariae* DNA or mixed DNA with *P. falciparum* 3D7 (*K13* wild-type).The LoDs of each duplex was investigated by using DNA extracted by the easyMAG^®^ system from artificial DBS containing decreasing parasite densities of each *k13* mutant (from 100 to 6.25 parasites/µL). The LoDs (> 95% detection in replicates) ranged from 50 (C580Y) to 25 (I543T, R539T, F446I) or 6.25 parasites/µL (Y493H). Data are shown in the Fig. 2a. The ranges of the mean Cq values according to the parasite density of *k13* mutants are displayed in the Fig. 2b (from 33.8 to 37.2 at 100 parasites/µL to 38.1–39.8 at 6.25 parasites/µL for the K13 mutant gene detection).

The reproducibility and the repeatability of the C580Y/Y493H and F446I/R539T duplexes were investigated. No significant differences in mean Cq values were observed according to the operators (N = 2, P = 0.82, F-test), days when the runs were performed (N = 2, P = 0.17, F-test), the easyMAG^®^ system used (N = 2, P = 0.83, F-test), or the batches of reagents tested (N = 2, P = 0.82, F-test).

A total of 284 clinical DBS were tested both at IP Cambodia (K13 reference assay) and at bioMérieux (K13 bMx prototype assay). The overall concordance between assays was excellent (97.5%, CI 95% 96.9–99.8%). Negative (no mutant) and positive (*k13* mutants included in the panel assay) concordances were 100% (CI 95% 96.5–100%) and 98.4% (CI 95% 94.3–99.6%), respectively. Only results from 7 DBS were found to be discordant (2.5%). The K13 bMx prototype misclassified two C580Y mutant samples as wild-type, four *P. falciparum* negative samples as F446I mutant (N = 1) or C580Y mutants (N = 3) and one F446I mutant sample as negative. Of note, seven *P. falciparum* samples harbouring *k13* mutations not included in the panel (A626E, C469F, G449A, P553L, P574L and Y511H) were correctly classified as no mutant by the K13 bMx prototype. All data are presented in Table 2.

**Table 2:**
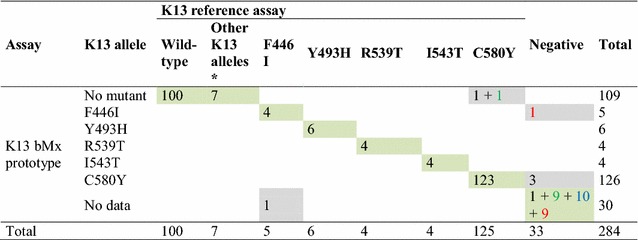
Evaluation of the analytical performances of the whole process (from DBS to results) of the K13 bMx prototype using 284 DBS collected in Cambodia and Myanmar and the K13 reference assay as gold standard method

* Other *k13* alleles include A626E, C469F, G449A, P553L, P574L and Y511H mutants

Cells coloured in green displayed concordant results and those coloured in grey discordant results. Numbers in black, green, red and blue fonts are results obtained from DBS collected from *P. falciparum* symptomatic malaria cases, *P. falciparum* asymptomatic carriers, *P. vivax* asymptomatic carriers, malaria-free individuals, respectively

### Clinical and analytical performances of the K13 bMx prototype (from DBS to results) performed in field conditions (step 4)

To evaluate the performances of the K13 bMx prototype in field conditions, 642 clinical DBS were tested with both assays at IP Cambodia. DBS included 390 specimens from Cambodia, 221 specimens from Myanmar and 31 specimens from Africa (Table 3).

**Table 3:** Origins of the DBS used to define the clinical performances of the K13 bMx prototype (from DBS to results) in field conditions

*Plasmodium* species	Clinical status	Asia		Africa	Total
		Cambodia	Myanmar		
–	Malaria-free	10			10
*P. falciparum*	Symptomatic	296	221	31	548
	Asymptomatic	63			63
*P. falciparum/P. vivax*	Symptomatic	17			17
	Asymptomatic	2			2
*P. vivax*	Symptomatic	1			1
	Asymptomatic	1			1
Total		390	221	31	642

Three technicians at IP Cambodia conducted the K13 bMx prototype assay and were blinded to the results of the K13 reference assay. Sequencing data were available for 606 samples, as presented in Table 4. The mutant I543T was not found in the set of clinical DBS. Among the 36 samples with no K13 sequencing valid data (10 malaria-free, 14 *P. falciparum* and 1 *P. vivax* infections from symptomatic patients and 10 *P. falciparum* and 1 *P. vivax* infections from asymptomatic individuals), the K13 bMx prototype assay detected 8 C580Y and 2 F446I mutants from *P. falciparum* symptomatic patients and 11 no mutant (4 from *P. falciparum* symptomatic patients, 5 from *P. falciparum* asymptomatic individuals, 1 from *P. vivax* symptomatic patient, 1 from *P. vivax* asymptomatic individual). The final analysis included only the 606 samples with available sequencing data.

**Table 4:** K13 sequencing data of 642 DBS tested with the K13 reference assay in field conditions

Sites	K13 reference assay	*Plasmodium* species				Total
		*P. falciparum*	*P. falciparum/P. vivax*	*P. vivax*	Negative	
ASIA	No data	21	3	2	10	36
	C580Y	244	12			256
	F446I	71				71
	R539T	21				21
	Y493H	20				20
	G538 V	1				1
	N458Y	8				8
	P553L	2				2
	P574L	1				1
	R561H	13				13
	S459L	1				1
	WT	177	4			181
	Total	580	19	2	10	611
AFRICA	V510V	1				1
	WT	30				30
	Total	31	0	0	0	31

The proportion of *k13* wild-type and mutants (PPs) among the 606 samples with available sequencing data were: wild-type or *k13* mutations not included in the panel assay (39.3%, 238/606), C580Y (42.2%, 256/606), F446I (11.7%, 71/606), R539T (3.5%, 21/606) and Y493H (3.3%, 20/606).

The overall concordance between results of both assays was 90.1% (546/606, CI 95% 82.7–98.0%), ranging from 90.0% (18/20, CI 95% 53.3–100%) (Y493H) to 95.8% (68/71, CI 95% 74.4–100%) (F446I) (Additional file 5). The concordance was higher using DBS from *P. falciparum* symptomatic patients (93.6%, 516/551, CI 95% 85.7–100.0%) compared to that obtained with DBS from *P. falciparum* asymptomatic individuals (54.5%, 30/55, CI 95% 36.8–77.9%, P = 0.02).

**Fig. 3:**
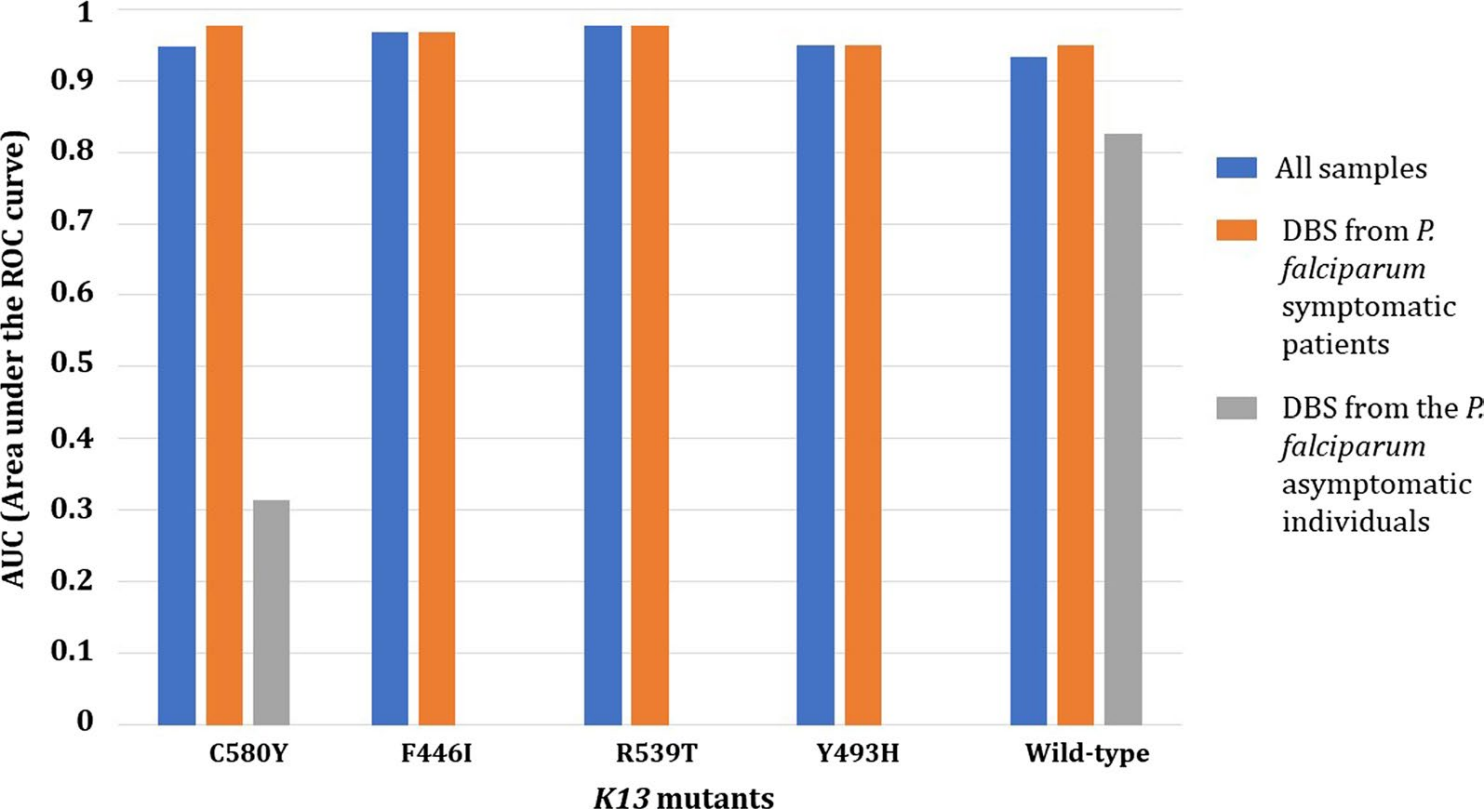
Area under the ROC curve values obtained from the K13 bMx prototype assay according to the *k13* mutant detected and the origin of the samples collected in Cambodia, Myanmar and Africa

By excluding the 16 DBS with low DNA yield (DBS classified as no valid data with the K13 bMx prototype assay), the final performances of the K13 bMx prototype assay were excellent, as shown in the Table 5. The sensitivity and specificity of the K13 bMx prototype assay were ≥ 90% (except for the detection of the C580Y in samples from asymptomatic individuals, sensitivity = 62.8%). Similarly, PPV and NPV were > 86% (except for the detection of wild-type in samples from asymptomatic individuals, PPV = 16.7%). As expected, due to the higher amount of DNA, the performances of K13 bMx prototype assay with DBS from *P. falciparum* symptomatic patients were better compared to those obtained with DBS collected from the *P. falciparum* asymptomatic individuals (Table 5). The areas under the ROC curves were estimated to be > 0.93 for all *k13* mutants in samples from symptomatic patients (Fig. 3). Raw data are presented in Additional files 5, 6 and 7.

**Table 5:** Summary of the performances of K13 bMx prototype according to the origin of the samples (all samples, N = 590; samples collected from *P. falciparum* symptomatic patients, N = 544; and samples collected from the *P. falciparum* asymptomatic individuals, N = 46)

Samples from	Performances	K13 mutants				K13 wild-type
		C580Y	F446I	R539T	Y493H	
All samples	Sensitivity (CI 95%)	90.2% (85.9–93.6)	95.8% (88.1–99.1)	95.2% (76.1–99.9)	90.0% (68.3–98.8)	94.1% (90.2–96.8)
	Specificity (CI 95%)	99.4% (97.6–99.9)	98.0% (96.2–99.0)	100% (99.4–100)	99.8% (99.0–99.9)	92.4% (89.2–94.9)
	AUC (CI 95%)	0.948 (0.927–0.965)	0.968 (0.951–0.981)	0.976 (0.961–0.987)	0.949 (0.928–0.965)	0.933 (0.909–0.952)
	PPV (CI 95%)	99.1% (96.6–99.8)	86.1% (77.5–91.7)	100%	94.7% (71.6–99.2)	88.2% (83.9–91.4)
	NPV (CI 95%)	93.0% (90.1–95.1)	99.4% (98.2–99.8)	99.8% (98.9–99.9)	99.7% (98.7–99.9)	96.3% (93.9–97.8)
*P. falciparum* symptomatic patients	Sensitivity (CI 95%)	95.8% (92.1–98.0)	95.8% (88.1–99.1)	95.2% (76.2–99.9)	90.0% (68.3–98.8)	94.0% (90.1–96.8)
	Specificity (CI 95%)	99.4% (97.8–99.9)	97.6% (95.7–98.8)	100% (99.3–100)	99.8% (98.9–99.9)	96.0% (93.3–97.9)
	AUC (CI 95%)	0.976 (0.958–0.987)	0.967 (0.949–0.981)	0.976 (0.960–0.987)	0.949 (0.927–0.966)	0.950 (0.929–0.967)
	PPV (CI 95%)	99.0% (96.2–99.8)	86.1% (77.5–91.7)	100%	94.7% (71.6–99.2)	94.1% (90.3–96.4)
	NPV (CI 95%)	97.3% (95.1–98.6)	99.3% (98.1–99.8)	99.8% (98.7–99.9)	99.6% (98.5–99.9)	96.0% (93.4–97.6)
*P. falciparum* asymptomatic individuals	Sensitivity (CI 95%)	62.8% (46.7–77.0)				100% (29.2–100)
	Specificity (CI 95%)	–				65.1% (49.1–79.0)
	AUC (CI 95%)	0.314 (0.185–0.468)				0.826 (0.699–0.915)
	PPV (CI 95%)	90.0% (87.7–91.9)				16.7% (11.7–23.1)
	NPV (CI 95%)	–				100%

*AUC* areas under the ROC curves, *PPV* positive predictive value, *NPV* negative predictive value, *CI 95%* confidence intervals 95%

## Discussion

Over a period of 18 months, the K13 bMx prototype was designed, optimized, developed and finally evaluated using the classical PCR and Sanger sequencing approach (K13 reference assay) as gold standard [15, 17]. The main objective of this project was to propose a robust and accurate field-based molecular assay to detect the five most prevalent validated *k13* mutations in an adapted throughput format. Results for 24 samples could be obtained in less than 4 h (including DNA extraction and real-time PCR runs) (Additional file 1). Quality assurance of the whole process was ensured by the semi-automated nucleic acid extraction platform from a new format of DBS (the most common matrix for blood sample collection used in surveillance surveys) and the inclusion of an internal control in the K13 bMx prototype assay.

Overall, the analytical and clinical performances of the K13 bMx prototype obtained from field clinical samples collected from symptomatic patients were excellent both in controlled (carried out at bioMerieux, Grenoble, France) and field conditions (carried out at IP Cambodia, Phnom Penh, Cambodia). However, those obtained from samples collected from asymptomatic individuals were largely lower. For instance, the K13 bMx prototype gave a result for almost all DBS from symptomatic patients tested in field conditions (544/551, 98.7% compared to 30/55, 54.5%, for DBS from asymptomatic individuals). The *k13* mutants were correctly detected: for C580Y in 95.8% (204/213 symptomatic patients) and 62.8% (27/43 asymptomatic individuals), for F446I in 95.8% (68/71), for the R539T in 95.2% (20/21) and for the Y493H in 90.0% (18/20). Wild-type allele or *k13* mutants not included in the panel assay were correctly classified as ‘no mutant’ in 91.1% (206/226) and 25.0% (3/12) in DBS collected from symptomatic patients and asymptomatic individuals, respectively.

The sensitivity and specificity of the K13 bMx prototype assay were ≥ 90%, except for samples collected from asymptomatic individuals. The sensitivity of the K13 bMx prototype to detect the C580Y mutant (the predominant allele in Southeast Asia) was significantly lower in assays using DBS collected from asymptomatic individuals (62.81%) compared to those performed with DBS from symptomatic patients (95.8%). This decrease in sensitivity was mainly due to the low amount of parasite DNA in DBS collected from asymptomatic individuals who were likely carrying parasites at densities lower than the LoD of the assay (ranging from 6.25 to 50 parasites/µL for the 5 mutants in the panel).

In field conditions, at IP Cambodia, the K13 ready-to-use bMx prototype assay was considered by the end-users as a user-friendly assay to perform. The interpretation of the results was facilitated by the design of an Excel file template. The end-users appreciated the shorter time to result than the K13 reference assay. The major advantages of the K13 bMx prototype over the K13 reference assay raised by the end-users were less requirement of budget planning (budget can be easily estimated per test) and fewer logistical issues linked to reagents purchase and the basic laboratory equipment needed to perform this assay. The findings seem to qualify the K13 bMx prototype assay as a screening tool usable in malaria endemic countries recognized to be at risk of spreading of ART-R parasites from Southeast Asia in their central laboratory dedicated to the monitoring of the anti-malarial drug resistance. However, rare occurrences of ART-R parasites that lack *k13* mutations with an increased RSA^0–3h^ survival rates or delayed parasite clearance times have been described [19, 33, 34] and suggest loci other than K13 may modulate ART-R. It seems, therefore, important to continue phenotypic assessment of ART-R, in addition to monitoring emergence of validated K13 mutants.

However, the K13 bMx prototype assay still has some limitations. First, the K13 bMx prototype assay has been developed to detect only 5 *k13* mutants. Nevertheless, the ARMS strategy and the flexible format allows us to update the K13 bMx prototype assay according to the epidemiological change (i.e. emergence of a novel and validated *k13* mutant) and to add new reactions detecting new *k13* mutants. Second, the K13 bMx prototype assay is restricted to the detection of ART-R parasites. However, based on the easyMAG^®^ platform and the Argene Solution^®^, the development of new field-based molecular assays detecting molecular signatures associated to anti-malarial drug resistances is feasible. New prototypes could include molecular markers associated with other anti-malarials used as ACT partner drugs (mefloquine, piperaquine) as well as molecular markers associated with anti-malarial drugs used in prophylaxis (sulfadoxine–pyrimethamine, atovaquone). To date molecular markers associated to amodiaquine or lumefantrine resistance are not well established, but marker panels could be adapted to detect mutations in *Pfcrt* and *Pfmdr1* genes.

Third, additional and multi-site studies are further needed to evaluate the performances of the K13 bMx prototype assay in field conditions, in different epidemiological contexts, especially settings recognized to be at risk of spreading of ART-R parasites from Southeast Asia (e.g. in Africa, India, South America). The ARTEMIS (for Artemisinin ResisTance fiEld MultIsite Study) project is planned to be conducted in 2018 to extend this first evaluation and to, generate additional evidences to consider the K13 bMx prototype assay a reference epidemiological field-based molecular tool for the global surveillance of ART-R.

## Additional files

**Additional file 1.** K13 bMx prototype assay 5-step workflow (24 DBS in less than 4 h).

**Additional file 2.** New 3 MM filter paper format design to facilitate the semi-automated extraction protocol and avoid inter-samples DNA contamination.

**Additional file 3.** Raw data showing the results between DBS tested at IP Cambodia using an in-house protocol (QiaAmp DNA blood mini kit Qiagen and two-step real-time PCR assays targeting the *Plasmodium cytochrome b* gene) and at bioMérieux using the easyMAG^®^ system (Generic 2.0.1 protocol) and the real-time PCR targeting the *cytochrome c oxidase subunit 1* gene (IC) according to the origin of the DBS.

**Additional file 4.** Principe of the Amplification-refractory mutation system (ARMS) strategy, based on allele-specific primers. In red is the mismatch position at the 3′ end of the primer. In blue an additional mismatch at the previous nucleotide.

**Additional file 5.** Overall clinical performances of the K13 reference assay obtained from 642 DBS collected from *P. falciparum* symptomatic patients in Cambodia, Myanmar and Africa and *P. falciparum* asymptomatic individuals in Cambodia. Cells coloured in green present concordant data obtained between the K13 bMx prototype assay and the K13 reference assay; Cells coloured in pale red present discordant data obtained between the K13 bMx prototype assay and the K13 reference assay.

**Additional file 6.** Clinical performances of the K13 reference assay obtained from 565 DBS collected from *P. falciparum* symptomatic patients in Cambodia, Myanmar and Africa. Cells coloured in green present concordant data obtained between the K13 bMx prototype assay and the K13 reference assay; Cells coloured in pale red present discordant data obtained between the K13 bMx prototype assay and the K13 reference assay.

**Additional file 7.** Clinical performances of the K13 reference assay obtained from 65 DBS collected from *P. falciparum* asymptomatic individuals in Cambodia. Cells coloured in green present concordant data obtained between the K13 bMx prototype assay and the K13 reference assay; Cells coloured in pale red present discordant data obtained between the K13 bMx prototype assay and the K13 reference assay.
