# Supplementary figures and images for: A novel field-based molecular assay to detect validated artemisinin-resistant k13 mutants
Source: Malar J. 2018 Apr 24;17:175. doi: 10.1186/s12936-018-2329-y (PMC5916714; doi:10.1186/s12936-018-2329-y)

**Additional File 1** K13 bMx prototype assay 5-step workflow (24 DBS in less than 4 hours)


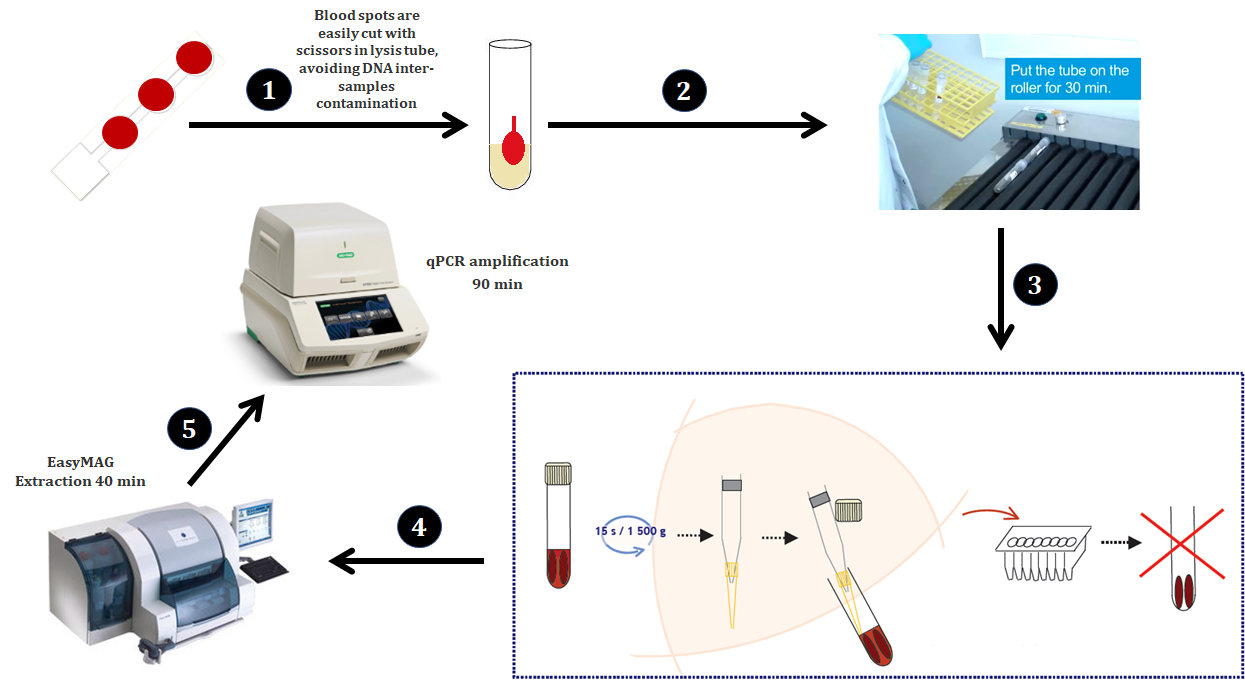

Supplement: Supplementary file 1 — Additional file 1. K13 bMx prototype assay 5-step workflow (24 DBS in less than 4 h). [file 12936_2018_2329_MOESM1_ESM.docx]
