# Supplementary material for: A novel field-based molecular assay to detect validated artemisinin-resistant k13 mutants
Source: Malar J. 2018 Apr 24;17:175. doi: 10.1186/s12936-018-2329-y (PMC5916714; doi:10.1186/s12936-018-2329-y)

**Additional File 2** New 3 MM filter paper format design to facilitate the semi-automated extraction protocol and avoid inter-samples DNA contamination.


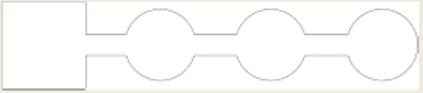

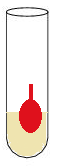


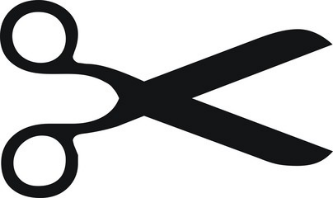

Supplement: Supplementary file 2 — Additional file 2. New 3 MM filter paper format design to facilitate the semi-automated extraction protocol and avoid inter-samples DNA contamination. [file 12936_2018_2329_MOESM2_ESM.docx]
