# Supplementary material for: A novel field-based molecular assay to detect validated artemisinin-resistant k13 mutants
Source: Malar J. 2018 Apr 24;17:175. doi: 10.1186/s12936-018-2329-y (PMC5916714; doi:10.1186/s12936-018-2329-y)

**Additional File 3** Raw data showing the results between DBS tested at IP Cambodia using an in-house protocol (QiaAmp DNA blood mini kit Qiagen and two-step real-time PCR assays targeting the *Plasmodium cytochrome b* gene) and at bioMérieux using the easyMAG^®^ system (Generic 2.0.1 protocol) and the real-time PCR targeting the *cytochrome c oxidase subunit 1* gene (IC) according to the origin of the DBS.


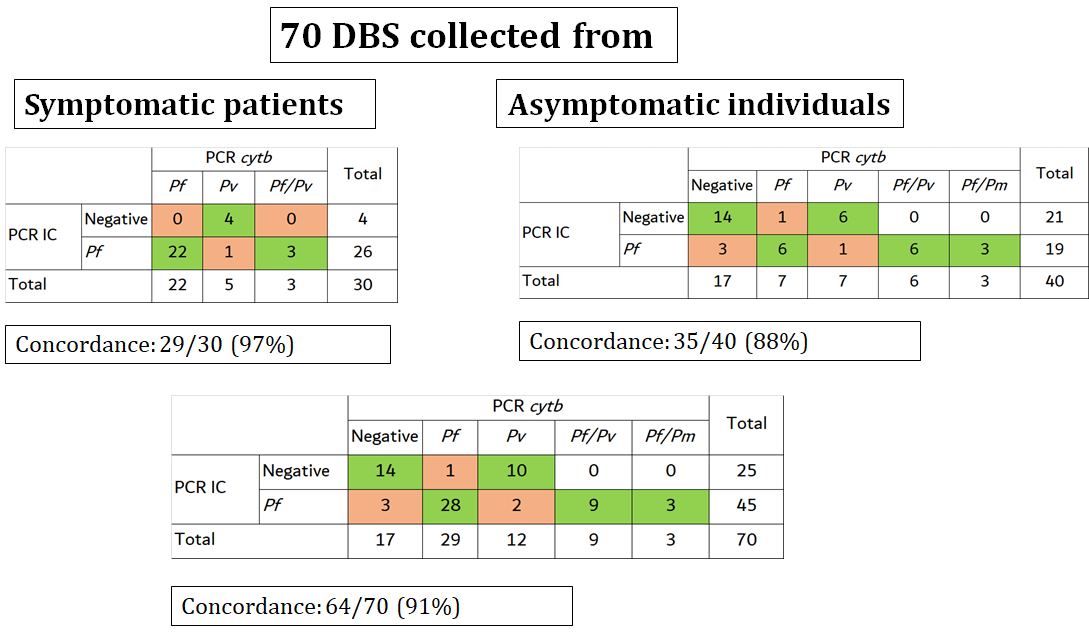

Supplement: Supplementary file 3 — Additional file 3. Raw data showing the results between DBS tested at IP Cambodia using an in-house protocol (QiaAmp DNA blood mini kit Qiagen and two-step real-time PCR assays targeting the Plasmodium cytochrome b gene) and at bioMérieux using the easyMAG® system (Generic 2.0.1 protocol) and the real-time PCR targeting the cytochrome c oxidase subunit 1 gene (IC) according to the origin of the DBS. [file 12936_2018_2329_MOESM3_ESM.docx]
