# Supplementary material for: A novel field-based molecular assay to detect validated artemisinin-resistant k13 mutants
Source: Malar J. 2018 Apr 24;17:175. doi: 10.1186/s12936-018-2329-y (PMC5916714; doi:10.1186/s12936-018-2329-y)
Supplement: Supplementary file 4 — Additional file 4. Principe of the Amplification-refractory mutation system (ARMS) strategy, based on allele-specific primers. In red is the mismatch position at the 3′ end of the primer. In blue an additional mismatch at the previous nucleotide. [file 12936_2018_2329_MOESM4_ESM.docx]

**Additional File 4** Principe of the Amplification-refractory mutation system (ARMS) strategy, based on allele-specific primers. In red is the mismatch position at the 3’ end of the primer. In blue an additional mismatch at the previous nucleotide.

In red is the mismatch position at the 3’ end of the primer. In green an additional mismatch at the previous nucleotide
