# Supplementary material for: A novel field-based molecular assay to detect validated artemisinin-resistant k13 mutants
Source: Malar J. 2018 Apr 24;17:175. doi: 10.1186/s12936-018-2329-y (PMC5916714; doi:10.1186/s12936-018-2329-y)
Supplement: Supplementary file 6 — Additional file 6. Clinical performances of the K13 reference assay obtained from 565 DBS collected from P. falciparum symptomatic patients in Cambodia, Myanmar and Africa. Cells coloured in green present concordant data obtained between the K13 bMx prototype assay and the K13 reference assay; Cells coloured in pale red present discordant data obtained between the K13 bMx prototype assay and the K13 reference assay. [file 12936_2018_2329_MOESM6_ESM.docx]

**Additional File 6** Clinical performances of the K13 reference assay obtained from 565 DBS collected from *P. falciparum* symptomatic patients in Cambodia, Myanmar and Africa. Cells coloured in green present concordant data obtained between the K13 bMx prototype assay and the K13 reference assay; Cells coloured in pale red present discordant data obtained between the K13 bMx prototype assay and the K13 reference assay.

| K13 reference assay | K13 bMx prototype assay | | | | | | | Total | % concordance |
| --- | --- | --- | --- | --- | --- | --- | --- | --- | --- |
|  | no data | no mutant | C580Y | F446I | R539T | Y493H | Y493H/R539T |  |  |
| No data |  | 4 | 8 | 2 |  |  |  | 14 |  |
| C580Y |  | 9 | 204 |  |  |  |  | 213 | 95.8% |
| F446I |  | 3 |  | 68 |  |  |  | 71 | 95.8% |
| R539T |  |  | 1 |  | 20 |  |  | 21 | 95.2% |
| Y493H |  | 1 |  |  |  | 18 | 1 | 20 | 90.0% |
| G538V |  | 1 |  |  |  |  |  | 1 | 91.2% |
| N458Y |  | 8 |  |  |  |  |  | 8 |  |
| P553L |  | 2 |  |  |  |  |  | 2 |  |
| P574L |  | 1 |  |  |  |  |  | 1 |  |
| R561H |  | 13 |  |  |  |  |  | 13 |  |
| S459L |  | 1 |  |  |  |  |  | 1 |  |
| WT | 7 | 179 | 1 | 11 |  | 1 |  | 199 |  |
| V510V |  | 1 |  |  |  |  |  | 1 |  |
| Total | 7 | 223 | 214 | 81 | 20 | 19 | 1 | 565 |  |
