# Supplementary material for: A novel field-based molecular assay to detect validated artemisinin-resistant k13 mutants
Source: Malar J. 2018 Apr 24;17:175. doi: 10.1186/s12936-018-2329-y (PMC5916714; doi:10.1186/s12936-018-2329-y)
Supplement: Supplementary file 7 — Additional file 7. Clinical performances of the K13 reference assay obtained from 65 DBS collected from P. falciparum asymptomatic individuals in Cambodia. Cells coloured in green present concordant data obtained between the K13 bMx prototype assay and the K13 reference assay; Cells coloured in pale red present discordant data obtained between the K13 bMx prototype assay and the K13 reference assay. [file 12936_2018_2329_MOESM7_ESM.docx]

**Additional File 7** Clinical performances of the K13 reference assay obtained from 65 DBS collected from *P. falciparum* asymptomatic individuals in Cambodia. Cells coloured in green present concordant data obtained between the K13 bMx prototype assay and the K13 reference assay; Cells coloured in pale red present discordant data obtained between the K13 bMx prototype assay and the K13 reference assay***.***

| K13 reference assay | K13 bMx prototype assay | | | | Total | % concordance |
| --- | --- | --- | --- | --- | --- | --- |
|  | no data | no mutant | C580Y | R539T/C580Y |  |  |
| No data | 5 | 5 |  |  | 10 |  |
| C580Y |  | 15 | 27 | 1 | 43 | 62.8% |
| WT | 9 | 3 |  |  | 12 | 25.0% |
| Total | 14 | 23 | 27 | 1 | 65 |  |
